# DAP3-mediated cell cycle regulation and its association with radioresistance in human lung adenocarcinoma cell lines

**DOI:** 10.1093/jrr/rrad016

**Published:** 2023-04-06

**Authors:** Yoshiaki Sato, Hironori Yoshino, Kota Sato, Ikuo Kashiwakura, Eichi Tsuruga

**Affiliations:** Department of Radiation Science, Hirosaki University Graduate School of Health Sciences, 66-1 Hon-cho, Hirosaki, Aomori 036-8564, Japan; Department of Radiation Science, Hirosaki University Graduate School of Health Sciences, 66-1 Hon-cho, Hirosaki, Aomori 036-8564, Japan; Department of Radiation Science, Hirosaki University Graduate School of Health Sciences, 66-1 Hon-cho, Hirosaki, Aomori 036-8564, Japan; Department of Radiation Science, Hirosaki University Graduate School of Health Sciences, 66-1 Hon-cho, Hirosaki, Aomori 036-8564, Japan; Department of Radiation Science, Hirosaki University Graduate School of Health Sciences, 66-1 Hon-cho, Hirosaki, Aomori 036-8564, Japan

**Keywords:** death-associated protein 3, radioresistance, G2 arrest, checkpoint kinase 1, lung adenocarcinoma, *in vitro*

## Abstract

Mitochondria play important roles in the cellular response to various types of stress, including that triggered by ionizing radiation. We have previously reported that the mitochondrial ribosomal protein death-associated protein 3 (DAP3) regulates the radioresistance of human lung adenocarcinoma (LUAD) cell lines A549 and H1299. However, the underlying mechanism of this regulation remains to be elucidated. To this end, we have herein investigated the role of DAP3 in the cell cycle regulation after irradiation. Notably, the DAP3 knockdown attenuated the radiation-induced increase of the G2/M cell population. Furthermore, western blotting analysis has revealed that the DAP3 knockdown decreased the expression of proteins related to the G2/M arrest, such as those of the phosphorylated cdc2 (Tyr15) and the phosphorylated checkpoint kinase 1 (Ser296), in irradiated A549 cells and H1299 cells. Moreover, by using a chk1 inhibitor, we were able to demonstrate that chk1 is involved in the radiation-induced G2/M arrest in both A549 and H1299 cells. Notably, the chk1 inhibitor was able to enhance the radiosensitivity of H1299 cells, while both chk1 inhibitor-abolished G2 arrest and inhibition of chk2-mediated events such as downregulation of radiation-induced p21 expression were required for enhancing radiosensitivity of A549 cells. Collectively, our findings reveal a novel role of DAP3 to regulate G2/M arrest through pchk1 in irradiated LUAD cells and suggest that chk1-mediated G2/M arrest regulates the radioresistance of H1299 cells, whereas both the chk1-mediated G2/M arrest and the chk2-mediated events contribute to the radioresistance of A549 cells.

## INTRODUCTION

Lung cancer is the leading cause of cancer-related deaths worldwide [1]. Non-small cell lung cancer accounts for 85% of lung cancer cases, of which lung adenocarcinoma (LUAD) is the largest subgroup [2]. Lung cancer can be treated through the use of chemotherapy, radiotherapy and surgery. However, the efficiency of radiotherapy can be stalled by radioresistance, thereby resulting in a reduced treatment success [3]. Hence, more light must be shed on the molecular mechanisms underlying the radioresistance of LUAD.

Mitochondria are cellular organelles responsible for energy conversion and ATP production in eukaryotic cells. Mitochondria have their own genome as well as a specific protein synthesis machinery served by the mitochondrial ribosomes. In addition to the ribosomal function, some of the mitochondrial ribosomal proteins are known to have their own specific roles in the other cellular functions [4–6]. For example, mitochondrial ribosomal protein L41 has been reported to block the anti-apoptotic activity of Bcl-2 and to induce caspase-mediated apoptosis [5]. In addition, Wang *et al*. have reported that mitochondrial ribosomal protein S16 promotes glioma cell growth, migration and invasion by the activating phosphatidylinositol-3 kinase/AKT/Snail axis. [6]. In terms of mitochondrial ribosomal protein S29 (death-associated protein 3, DAP3), Henning has reported that the overexpression DAP3 can confer radioresistance to ataxia-telangiectasia cells exhibiting high radiosensitivity [7]. We have also reported that DAP3 is involved in the radioresistance of human LUAD cell lines [8], though it remains unclear how DAP3 can modulate the radioresistance.

Ionizing radiation is known to cause cytotoxicity through DNA damage. In irradiated cells, cell cycle progression is transiently arrested for the undertaking of DNA repair through the activation of checkpoint kinases, thereby resulting in radioresistance [9, 10]. Ionizing radiation is known to activate checkpoint kinase 1 (chk1) and lead to cell cycle G2/M arrest through the inactivation of cyclin B1 and the cdc2 complex, whereas chk2 activation leads to a cell cycle G1 arrest through p53 [10, 11]. It has also been reported that some proteins involved in resistance to chemotherapy and radiotherapy actually regulate the cell cycle arrest [12, 13]. For example, Huang *et al*. have reported that the Ras-associated binding protein Rab12 mRNA and protein expressions are upregulated in cervical cancer tissues, and that Rab12 promotes radioresistance by inducing a G2/M arrest [13]. Therefore, we have hypothesized that DAP3 can regulate radioresistance through a cell cycle regulation. However, to date, there have been no reports regarding this issue. Therefore, this study has investigated our hypothesis that DAP3 regulates radioresistance through radiation-induced cell cycle arrest in human LUAD cell lines.

## MATERIALS AND METHODS

### Reagents

Calcium- and magnesium-free phosphate-buffered saline PBS(−) and paclitaxel (#169-18 611) were purchased from Wako Pure Chemical Industries, Ltd (Osaka, Japan). Propidium iodide (PI) and the chk2 inhibitor II were purchased from Sigma-Aldrich (Merck KGaA, Darmstadt, Germany). SCH900776 was purchased from Med Chem Express (Shanghai, China). The anti-rabbit horseradish peroxidase (HRP)-conjugated IgG (#7074), and the anti-mouse HRP-conjugated IgG (#7076) secondary antibodies, as well as the anti-phospho-cdc2 (Tyr15) (#9111), the anti-cyclin B1 (#4135), the anti-phospho-histone H3 (Ser10) XP (#3377), the anti-phospho-chk1 (Ser296) (#2349), the anti-chk1 (#2360), the anti-phospho-chk2 (Thr68) (#2661), the anti-p21 (#2947) and the anti-β-actin (#4967) monoclonal antibodies were purchased from Cell Signaling Technology Inc (Danvers, MA, USA). The anti-DAP3 (Cat. No. 610662) monoclonal primary antibody was purchased from BD Biosciences (Franklin Lakes, NJ, USA). The Ambion Silencer® Select Pre-designed siRNA against the gene-encoding DAP3 (Cat. No. s1506) and the Silencer® Select Negative #1 Control (Cat. No. AM4611) siRNAs were purchased from Thermo Fisher Scientific, Inc (Waltham, MA, USA).

### Cell culture and treatment

Human LUAD A549 and H1299 cells were purchased from the Riken Bio-Resource Center (Tsukuba, Japan) and the American Type Culture Collection (Manassas, VA, USA), respectively. A549 cells were maintained in Dulbecco’s modified Eagle’s medium (Sigma-Aldrich) supplemented with 10% heat-inactivated fetal bovine serum (FBS; Sigma-Aldrich) and 1% penicillin/streptomycin (P/S; Wako Pure Chemical Industries, Ltd), at 37°C, in a humidified atmosphere of 5% CO_2_. H1299 cells were maintained in RPMI 1640 medium (Gibco®; Invitrogen/Thermo Fisher Scientific, Waltham, MA, USA) and supplemented with 10% FBS and 1% P/S, at 37°C, in a humidified atmosphere of 5% CO_2_.

Cells were seeded onto 35 mm culture dishes (6.0 × 10^4^ cells) or 60 mm culture dishes (1.2 × 10^5^ cells) (Sumitomo Bakelite Co., Ltd, Tokyo, Japan) and were cultured overnight so as to allow them to adhere to the dish. Subsequently, the cells were harvested by using 0.1% trypsin-ethylenediaminetetraacetic acid (Wako Pure Chemical Industries, Ltd), and the number of viable cells was counted by using a trypan blue dye exclusion assay before any subsequent analysis was undertaken. In experiments using checkpoint kinase inhibitors, we used SCH900776 as a chk1 inhibitor [14] and chk2 inhibitor II as a chk2 inhibitor [15]. In fact, chk1 (400 nM) and/or chk2 (10 μM) inhibitors were added to the culture medium just 1 h before the irradiation.

### *In vitro* X-ray irradiation

Cells were irradiated (150 kVp; 20 mA; 0.5 mm Al filter and 0.3 mm Cu filter) by using an X-ray generator (MBR-1520R-3; Hitachi, Ltd, Tokyo, Japan) at a distance of 450 mm from the focus and at a dose rate of 1.00–1.03 Gy/min.

### siRNA transfection

Transfections of siRNA targeting either DAP3 or the control siRNA were performed twice by using Lipofectamine® RNAiMAX (Invitrogen; Thermo Fisher Scientific, Inc) according to the manufacturer’s protocol. In brief, cells transfected for 48 h were harvested, transfected again and cultured for another 48 h. After the second transfection, the cells were harvested and used for the undertaking of subsequent analyses. The final concentration of all siRNAs was 10 nM.

### Cell cycle analysis

Cell cycle analysis was performed, as previously described [16]. Harvested cells were fixed overnight in ice-cold 70% ethanol at −20°C. Fixed cells were washed with and subsequently suspended in PBS(−), and they were treated with 20 μg/ml RNase A for 30 min, at 37°C. Following this treatment, the cells were resuspended in PBS(−) containing 20 μg/ml PI and were incubated in the dark for 30 min. Finally, the cells were passed through a cell strainer (BD Falcon; BD Biosciences, Franklin Lakes, NJ, USA) and were analyzed cells (10 000 cells/sample) by using a flow cytometer (Cytomics FC500 with CXP software; Beckman–Coulter, Fullerton, CA, USA).

### SDS-PAGE and western blotting

SDS-PAGE and western blotting analysis were performed as previously reported [17]. The following primary antibodies were used: anti-phospho-cdc2 (1:3000), anti-cyclin B1 (1:3000), anti-phospho-histone H3 (1:3000), anti-cyclin B1 (1:3000), anti-phospho-chk1 (1:3000), anti-chk1 (1:3000), anti-phospho-chk2 (1:3000), anti-DAP3 (1:3000), anti-p21 (1:3000), anti-GAPDH (1:4000) and anti-β-actin (1:4000). The following secondary antibodies were used: HRP-conjugated anti-rabbit IgG (1:10 000) and HRP-conjugated anti-rabbit IgG (1:10 000). The antigens were visualized by using the Clarity MAX™ Western ECL Substrate (Bio-Rad Laboratories, Inc, Hercules, CA, USA) for phosphor-chk2 or the Clarity™ Western ECL Substrate (Bio-Rad Laboratories, Inc) for the other proteins. Blot stripping was performed by using Stripping Solution (Wako Pure Chemical Industries, Ltd). Images for proteins other than p21 were captured by using the Cool Saver AE-6955 (ATTO, Tokyo, Japan), whereas the images of p21 were captured by using the iBright 1500 Imaging System (Invitrogen; Thermo Fisher Scientific, Inc). The quantification of the bands was performed by using the ImageJ software (National Institutes of Health, Bethesda, MD, USA).

### Mitotic catastrophe analysis

The cells on coverslips were incubated under the indicated conditions, were fixed for 10 min in 4% paraformaldehyde in PBS(−), and were then permeabilized with 1% Triton X-100 in PBS(−) for 10 min, at room temperature. After 24 h, the cells were harvested for the undertaking of the subsequent analysis. The samples were mounted onto coverslips by using Vectashield® Mounting Medium with DAPI (Vector Laboratories, Inc, Burlingame, CA, USA) and were examined by using an Olympus IX71 fluorescent microscope (Tokyo, Japan) and the DP2-BSWsoftware (Olympus). At least 100 cells were analyzed.

### Clonogenic survival assay

Cells were seeded onto 35 mm culture dishes (6.0 × 10^4^ cells) and were incubated overnight. After incubation, the cells were exposed to X-rays and were further incubated for 24 h. The cultured cells were harvested by using 0.1% trypsin-ethylenediaminetetraacetic acid and were seeded onto 60 mm culture dishes. The cells were further incubated for 7–8 days, were fixed with methanol and were stained with Giemsa solution (Wako Pure Chemical Industries, Ltd). Experiments were performed in triplicate. Colonies containing >50 cells were counted. The surviving fraction at each radiation dose was calculated as previously described in detail [18].

### Statistical analysis

Data are presented as the mean ± standard deviation (SD) of three independent experiments. Comparisons between the control and experimental groups were performed by using the two-sided Student’s *t*-test or a Mann–Whitney U-test depending on data distribution. Values of *P* found to be <0.05 were considered as indicative of statistically significant differences. The Excel 2016 software (Microsoft, Washington, DC, USA) and the add-in software Statcel 4 (The Publisher OMS Ltd, Tokyo, Japan) were used to perform the required statistical analyses. When the control group was considered as 100%, one sample *t*-test was performed by using GraphPad QuickCalcs (https://www.graphpad.com/quickcalcs/).

## RESULTS

### Involvement of DAP3 in radiation-induced G2/M arrest

**Fig. 1:**
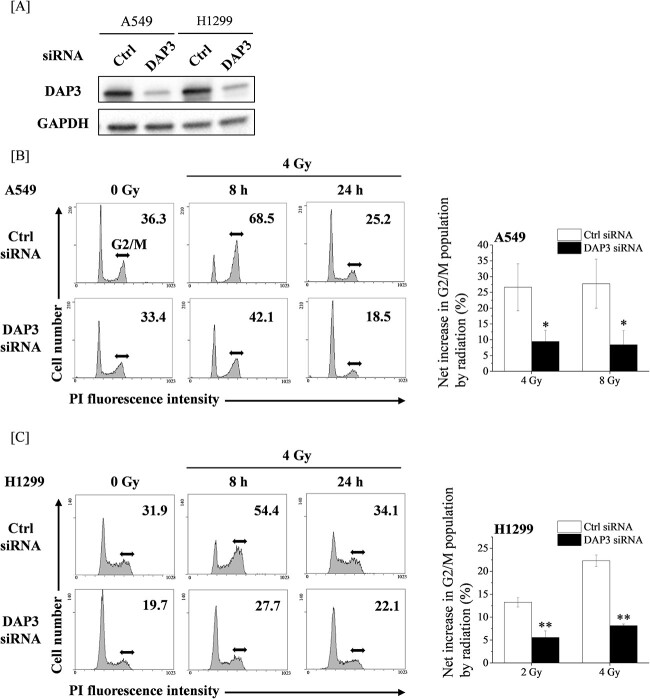
Involvement of DAP3 in the radiation-induced G2/M arrest of human LUAD cell lines. (**A**) Human LUAD cell lines (A549 and H1299) transfected with control or DAP3 siRNA were harvested, and the DAP3 protein expression was analyzed through western blotting. A representative image of an immunoblot is shown. GAPDH was used as the loading control. (**B**, **C**) Human LUAD cell lines transfected with control or DAP3 siRNA were treated with radiation. After 8 or 24 h of culturing, the cells were harvested for cell cycle analysis. (Left) Representative histograms and the obtained data are presented. Double-headed arrows indicate the G2/M population, whereas the inset number in the figure indicates the G2/M proportion in the total cells. (Right) The net increase in G2/M populations as a result of the undertaken irradiation is shown. Data are presented as the mean ± SD of three independent experiments. Symbols used: ^*^P < 0.05 and ^*^^*^P < 0.01; both versus control siRNA.

We initially investigated the cell cycle distribution of DAP3-knockdown A549 and H1299 cells (Fig. 1A). As shown in Fig. 1B, although the DAP3 knockdown hardly affected the cell cycle distribution of non-irradiated A549 cells, it did attenuate the increase in G2/M population at 8 h after irradiation. Similar results were observed in H1299 cells (Fig. 1C). These results suggest that DAP3 is involved in the radiation-induced G2/M arrest in human LUAD cell lines.

### Enhancement of radiation-induced mitotic catastrophe by DAP3 knockdown

**Fig. 2:**
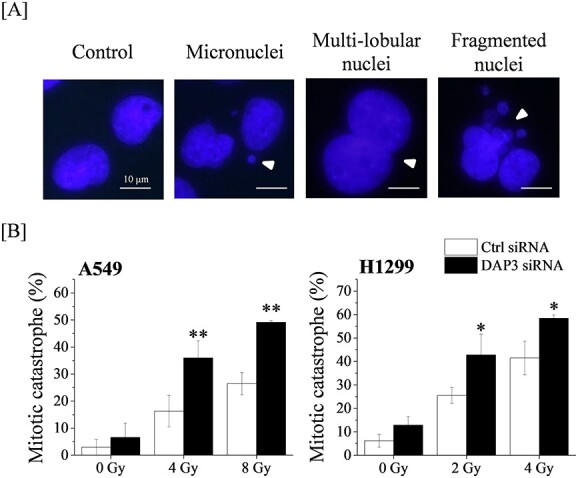
Effect of DAP3 knockdown on radiation-induced mitotic catastrophe in human LUAD cell lines. (**A**, **B**) Human LUAD cell lines transfected with control or DAP3 siRNA were treated with radiation. After 72 h of culturing, the cells were harvested for the analysis of mitotic catastrophe. (Top) Representative pictures of mitotic catastrophe are shown. Micronuclei, multilobed nuclei and fragmented nuclei were counted as indicative of the occurring mitotic catastrophe. Arrows indicate the cells undergoing mitotic catastrophe. (B) The percentages of the cells affected by mitotic catastrophe are shown. Data are presented as the mean ± SD of three independent experiments. Symbols used: ^*^P < 0.05 and ^*^^*^P < 0.01; both versus control siRNA.

Since the aberrant cell cycle checkpoints usually result in mitotic catastrophe, which is a form of radiation-induced cell death [19–21], we subsequently examined the effect of DAP3 knockdown on radiation-induced mitotic catastrophe. To identify mitotic catastrophe, the cells were analyzed for the presence of micronuclei, multi-lobular nuclei, and fragmented nuclei whose nucleus has split into more than three fragments, or disintegrated into many tiny fragments (Fig. 2A). As shown in Fig. 2B, DAP3 knockdown hardly increased the percentage of mitotic catastrophe in non-irradiated cells. As expected, the percentage of mitotic catastrophe was significantly higher in DAP3-knockdown-irradiated cells than that in control-irradiated cells.

### Reduction of radiation-induced expressions of proteins related to G2/M arrest by DAP3 knockdown

**Fig. 3:**
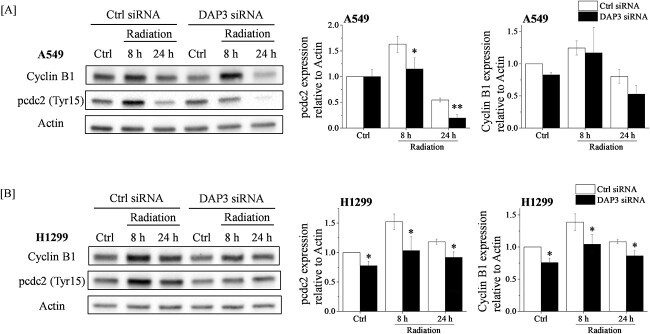
Effects of DAP3 knockdown on the expression of G2 arrest regulators in irradiated human LUAD cell lines. (**A**, **B**) A549 (A) and H1299 (B) cells transfected with control or DAP3 siRNA were treated with 4 Gy irradiation and were cultured for 8 or 24 h. The cells were harvested for the undertaking of western blot analysis. A representative image of an immunoblot is shown. Actin was used as the loading control. The relative values of the pcdc2/actin and the cyclin B1/actin ratios are presented, where pcdc2 indicates the phosphorylated-cdc2. Data are presented as the mean ± SD of three independent experiments. Symbols used: ^*^P < 0.05 and ^*^^*^P < 0.01; both versus control siRNA.

In an attempt to elucidate the role of DAP3 in radiation-induced G2/M arrest, we first investigated whether the radiation-induced G2/M arrest is a G2- or an M-phase arrest through the study of the expression of phosphorylated-histone H3 (pH 3), which is known to be upregulated in mitotic cells. We used paclitaxel-treated A549 cells as a positive control for M-phase cells where an upregulation of the pH 3 expression was evident (Supplementary Fig. 1), whereas a lower expression of pH 3 was observed in radiation-induced G2/M arrested A549 cells, and DAP3 knockdown hardly affected the pH 3 expression in irradiated cells (Supplementary Fig. 1). These findings indicate that the cell cycle arrest occurred at the G2 phase (rather than the M-phase) in the irradiated cells, and that DAP3 regulates the radiation-induced G2 arrest in human LUAD cell lines. Therefore, we then investigated the effect of the DAP3 knockdown on the expression of key regulators of the G2 arrest. The latter is known to be accompanied by an upregulation of cyclin B1 and a phosphorylation of cdc2 (pcdc2) [10, 11]. Figure 3A shows that the expressions of cyclin B1 and pcdc2 were increased in the irradiated A549 cells, and that the DAP3 knockdown decreased the radiation-induced increase of the pcdc2 expression. In H1299 cells, DAP3 knockdown attenuated the steady level as well as the radiation-induced expression of both cyclin B1 and pcdc2 (Fig. 3B).

### Involvement of DAP3 in radiation-induced phosphorylated expressions of checkpoint kinases

**Fig. 4:**
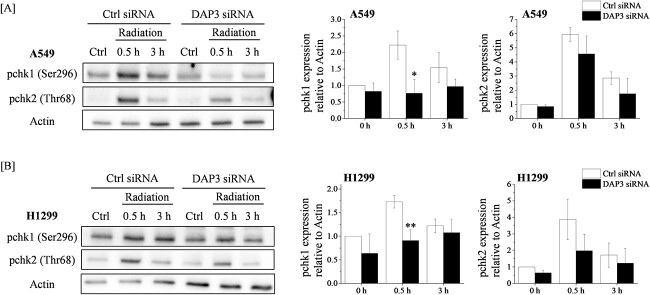
Effects of DAP3 knockdown on the expression of checkpoint kinases in irradiated human LUAD cell lines. (**A**, **B**) A549 (A) and H1299 (B) cells transfected with control or DAP3 siRNA were treated with 4 Gy irradiation and were cultured for 0.5 or 3 h. The cells were harvested for the undertaking of western blot analysis. A representative image of an immunoblot is shown. Actin was used as the loading control. The relative values of the pchk1/actin and the pchk2/actin ratios are presented, where pchk1 and pchk2 indicate the phosphorylated-chk1 and the phosphorylated-chk2, respectively. Data are presented as the mean ± SD of three independent experiments. Symbols used: ^*^P < 0.05 and ^*^P < 0.01; both versus control siRNA.

To further characterize the underlying mechanism of the DAP3-mediated radiation-induced G2 arrest, we investigated the effect of DAP3 knockdown on the expression of chk1 and of chk2, which are regulators of cdc2 and cyclin B1 [10, 11]. As shown in Fig. 4A, the phosphorylated checkpoint kinase 1 (pchk1)/actin and the pchk2/actin ratios were increased at 0.5 h after the irradiation. Interestingly, the DAP3 knockdown significantly suppressed the increase in the pchk1/actin ratio, and partially suppressed the pchk2/actin ratio in irradiated cells (Fig. 4A). Similar effect of DAP3 knockdown on pchk1 and pchk2 expressions was observed in H1299 cells (Fig. 4B). In addition, we found that although radiation hardly affected total chk1 expression in control cells, knockdown of DAP3 significantly decreased total chk1 expressions in A549 cells (Supplementary Fig. 2).

### Involvement of chk1 in radiation-induced G2 arrest

**Fig. 5:**
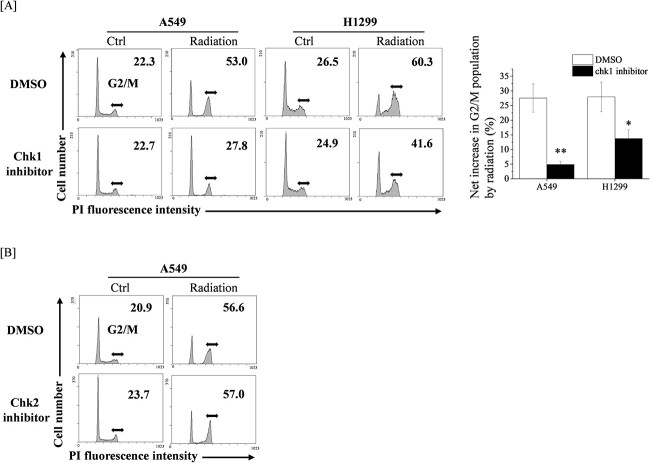
Involvement of chk1 in radiation-induced G2/M arrest in human LUAD cell lines. (**A**) Human LUAD cell lines were incubated with DMSO or with a chk1 inhibitor. After incubation for 1 h, the cells were irradiated (4 Gy). After 8 h of culturing, the cells were harvested for the undertaking of cell cycle analysis. (Left) Representative histograms and the obtained data are presented. Double-headed arrows indicate the G2/M population, whereas the inset number in the figure indicates the G2/M proportion in the total cells. (Right) The net increase in G2/M populations as a result of the undertaken irradiation is shown. Data are presented as the mean ± SD of three independent experiments. Symbols used: ^*^P < 0.05 and ^*^^*^P < 0.01; both versus DMSO. (**B**) A549 cells treated with DMSO or with a chk2 inhibitor were cultured for 8 h. The cells were harvested for the undertaking of cell cycle analysis. Representative histograms and the obtained data are presented. Double-headed arrows indicate the G2/M population, whereas the inset number in the figure indicates the G2/M proportion in the total cells.

Since the DAP3 knockdown decreased the radiation-induced pchk1 expression in both A549 and H1299 cells (Fig. 4), we subsequently examined the role of chk1 on the radiation-induced G2 arrest in human LUAD cell lines by using the chk1 inhibitor. As shown in Fig. 5A, similarly to the DAP3 knockdown, chk1 inhibitor decrease the radiation-induced G2/M population in human LUAD cell lines. In contrast, the chk2 inhibitor hardly affected the radiation-induced G2 population in A549 cells (Fig. 5B).

### Involvement of checkpoint kinases in the regulation of radioresistance

**Fig. 6:**
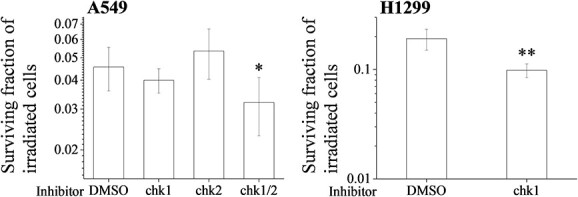
Involvement of checkpoint kinases in the radioresistance of human LUAD cell lines. (Left) A549 cells were incubated with DMSO or with a chk1 and/or a chk2 inhibitor and (Right) H1299 cells were incubated with DMSO or with a chk1 inhibitor. After an incubation for 1 h, the cells were irradiated (6 Gy). After 24 h of culturing, the cells were harvested and seeded for the undertaking of colony formation assays. The results are shown as the surviving fraction of human LUAD cell lines. Data are presented as the mean ± SD of three independent experiments. Symbols used: ^*^P < 0.05 and ^*^^*^P < 0.01; both versus DMSO.

Since we found that chk1 is essential for the radiation-induced G2 arrest (Fig. 5A), we investigated whether chk1 is involved in the radioresistance of human LUAD cell lines. As shown in Fig. 6, the chk1 inhibitor was able to decrease the surviving fraction of the irradiated H1299 cells (when compared with the DMSO treatment), whereas there was no significant difference between the DMSO- and the chk1 inhibitor-treated irradiated A549 cells. Although the chk2 inhibitor alone also failed to decrease the surviving fraction of the irradiated A549 cells, a cotreatment with the chk1 and the chk2 inhibitors significantly decreased the surviving fraction of the irradiated A549 cells (Fig. 6, left). These findings suggest that the chk1-mediated G2 arrest contributes to the radioresistance of H1299 cells, whereas the chk2-mediated events as well as the chk1-mediated G2 arrest are involved in the radioresistance of the A549 cells.

### Reduction of the radiation-induced p21 expression by the DAP3 knockdown

**Fig. 7:**
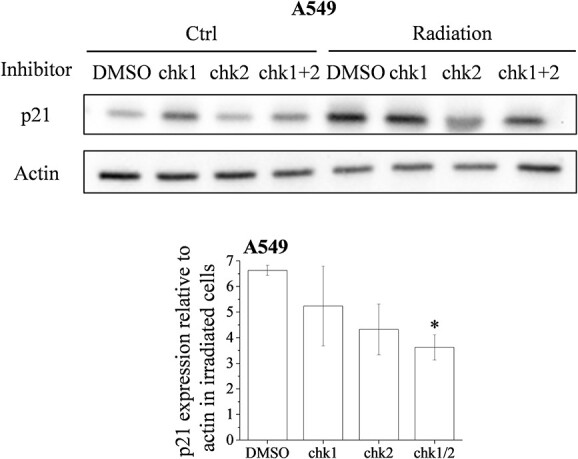
Effects of checkpoint kinase inhibitors on the radiation-induced p21 expression in A549 cells. A549 cells were incubated with DMSO or with a chk1 and/or a chk2 inhibitor. After an incubation for 1 h, the cells were irradiated (8 Gy). The cells were harvested for the undertaking of western blot analysis. A representative image of an immunoblot is shown. Actin was used as the loading control. The relative value of the p21/actin ratio is presented. Data are presented as the mean ± SD of three independent experiments. Symbols used: ^*^P < 0.05 and ^*^^*^P < 0.01; both versus DMSO.

We finally explored the chk2-mediated events that can potentially contribute to the radioresistance of A549 cells. We have, herein, focused on p21, which is known to regulate radioresistance and be induced by a chk2-mediated pathway [11, 22, 23]. As shown in Fig. 7, the expression of p21 was increased at 24 h after irradiation. Notably, a cotreatment with the chk1 and the chk2 inhibitors resulted in a decrease of the radiation-induced p21 expression.

## DISCUSSION

DAP3 is known as a mediator of apoptosis induced by interferon-gamma, the Fas ligand and the tumor necrosis factor alpha [24, 25]. However, recent studies have revealed a pro-survival and oncogenic function of DAP3 [26, 27]. For example, Wazir *et al*. have recently reported that a DAP3 knockdown can inhibit the growth of breast cancer cells [26]. In addition, Han *et al*. have shown that the subcutaneous injection of a DAP3-depleted esophageal squamous cell carcinoma cell line into mice can lead to the formation of much smaller tumors (when compared with those formed by the injection of control cells) [27]. An analysis using a public database has revealed that the expression levels of the DAP3 mRNA in LUAD tissue are significantly higher than those in normal tissue (Supplementary Fig. 3A), and that LUAD patients with high mRNA expressions of DAP3 have poor outcomes (Supplementary Fig. 3B). In addition, DAP3 knockdown A549 and H1299 cells display a lower ability of colony formation compared with control cells (Supplementary Fig. 3C). Taken together, these findings suggest that DAP3 could serve as a potential therapeutic target for LUAD as well as for other tumors.

Recent studies have shown that many cancer cells rely on mitochondrial respiration to fuel tumorigenesis [28]. For example, LUAD harbors frequent mutations in some components of chromatin-remodeling complex causing upregulation of oxidative phosphorylation (OXPHOS), and conferring sensitivity to OXPHOS inhibition [28]. DAP3 is one of the components of mitochondrial ribosomes, which synthesize proteins of the mitochondrial respiratory chain [4], and DAP3 knockdown is reported to impair this protein synthesis [29]. Therefore, there is a possibility that DAP3 is involved in the proliferation of A549 and H1299 cells through the synthesis of mitochondrial respiratory chain proteins.

Although our previous report has shown that DAP3 could be an effective target for the improvement of the radiosensitivity of human LUAD cells [8], the molecular mechanisms underlying the DAP3-mediated radioresistance of LUAD cells remain unclear. Hence, we have herein investigated the involvement of DAP3 in the radiation-induced cell cycle arrest, as well as its role in the radioresistance of human LUAD cell lines. Our findings demonstrate that the DAP3 knockdown decreases the radiation-induced G2 arrest and the expression of proteins related to the cell cycle arrest such as pcdc2 and pchk1. In addition, our experiments with the use of chk1 and chk2 inhibitors have shown that chk1 regulates the radioresistance of H1299 cells through a G2 arrest, whereas both the chk1-mediated G2 arrest and the chk2-mediated events may contribute to the radioresistance of A549 cells.

DAP3 is well-known to be involved in the mitochondrial ribosomal function, cell death and RNA editing [24, 25, 27, 29]. As far as we know, this is the first study demonstrating that DAP3 is involved in the radiation-induced G2 arrest through the phosphorylation of chk1, despite the fact that it remains unclear how DAP3 regulates the radiation-induced pchk1. Since ATM as well as ATM and RAD3-related (ATR) participate in the regulation of chk1 and chk2 phosphorylation [30], it is possible that DAP3 might control the radiation-induced pchk1 through the regulation of ATM or of ATR. In addition, there are other possibilities in the regulation of radiation-induced pchk1 by DAP3. For instance, the mitochondrial ribosomal function of DAP3 (i.e. the ATP production) is involved in the regulation of radiation-induced pchk1, as the ATP is essential for phosphorylation and the DAP3 knockdown is known to decrease the ATP production [29]. Furthermore, since DAP3 knockdown also decreased total chk1 expression (Supplementary Fig. 2), DAP3 may regulate pchk1 expression through the phosphorylation process and/or total protein expression. Therefore, further studies are required to explore these possibilities.

DAP3 knockdown decreased the population of G2/M in non-irradiated H1299 cells (Fig. 1C). It is known that G2 arrest resulted from a downregulation of the processes that activate cdc2 [31]. In addition, Patrick *et al*. reported that G2 arrest correlated with the accumulation of tyrosine-phosphorylated cdc2 [32]. Moreover, Winters *et al*. have reported that gamma-irradiation induced G2 arrest with cdc2 extensively phosphorylated at the inhibitory sites Thr14 and Tyr15 in H1299 cells [33]. Since DAP3 knockdown decreased the steady-state level of pcdc2 in H1299 cells (Fig. 3B), it might cause the decrease in G2/M population in DAP3-knockdown H1299 cells.

As shown in Fig. 3A, the expression of pcdc2 of 24 h after irradiation was low both in control and DAP3 siRNA transfected A549 cells. It is known that pcdc2 on Tyr15 inhibits its activity during G2 phase of the cell cycle, whereas de-phosphorylation on Tyr15 occurred during early mitosis [31]. Since most of the A549 cells have released from G2 arrest at 24 h after 4 Gy irradiation (Fig. 1B), it is thought that low expression of pcdc2 at 24 h after 4 Gy irradiation may be due to low G2/M population at the time.

Cell cycle checkpoints are promising targets for the sensitization of cancer cells to radiation, and many studies have shown that by abrogating a G2 arrest one can sensitize a panel of human cancer cells to radiation [34–36]. For example, Patel *et al*. have shown that the radiosensitizing effect of the chk1 inhibitor CCT244747 was elicited by the abrogation of the radiation-induced G2 arrest [35]. In addition, Liu *et al*. have revealed a negative correlation of the cellular radiosensitivity with the accumulated the G2/M arrested cells [37]. Therefore, it is likely that the DAP3 knockdown-induced abrogation of G2 arrest might at least be partially involved in the enhancement of radiosensitivity induced by the DAP3 knockdown.

We have, herein, also identified a differential effect of the chk1 inhibitor on the radiosensitivity of A549 and of H1299 cells. Bridge *et al*. have reported that MK-8776 (another name for the chk1 inhibitor used in this study) was able to radiosensitize p53-defective cancer cell lines, but not cell lines with wild-type p53, deriving from human non-small cell lung cancer and human head and neck squamous cell carcinomas [36]. Furthermore, Borst *et al*. have reported that the chk1 inhibitor SAR020106 can enhance radiosensitivity in p53-deficient Cal27, HN6 and HeLa cells, but not in p53 wild-type A549 cells [38]. p53 is a transcription factor that regulates many biological pathways, including those involved in DNA repair as well as the chk1-mediated pathway leading to cell cycle arrest [10, 39]. Since A549 cells contain wild-type p53 [38], whereas H1299 cells contain no p53 gene [36], the different effect of the chk1 inhibitor on the radiosensitivity of A549 and of H1299 cells may be attributed to the difference in terms of their p53 status.

It is well known that chk2 regulates p53-related pathways such as p21-mediated pathway, and p21 is involved in the radioresistance of various tumor types, including some of those occurring in the lungs, the brain, the prostate, the cervix, the esophagus, and the large intestine, as well as in nasopharyngeal carcinoma. For example, p53-p21 is known to mediate cell cycle G1 arrest, senescence and glycolysis under hypoxia [23]. Since a cotreatment with a chk1 and a chk2 inhibitors can enhance the radiosensitivity of A549 cells along with the induction of a decrease in radiation-induced p21 expression, it is possible that p21 might be involved in DAP3-mediated radioresistance. Of course, one cannot exclude the possibility that factors other than p21 might contribute to the DAP3-mediated radioresistance, as the p53-chk2 pathway regulates many signaling pathways [23].

## CONCLUSION

In conclusion, our findings reveal a novel role of DAP3 to regulate G2 arrest through pchk1 in irradiated LUAD cells. In addition, the present results suggested that a G2 arrest can regulate the radioresistance of H1299 cells, whereas both the chk1-mediated G2 arrest and the chk2-mediated events may contribute to the radioresistance of A549 cells. We hope that these findings related to radioresistance might improve the efficacy of radiotherapy for human LUAD.

## DATA AVAILABILITY

The data presented in this study are available in the article.

## FUNDING

This work was supported by JST SPRING (grant number: JPMJSP2152) and partially supported by JSPS KAKENHI (grant number: JP21K07691).

## CONFLICT OF INTEREST

There are no conflicts of interest to declare.

## Supplementary Material

Figure_s1_rrad016

Figure_S2_revised_rrad016

Figure_S3_revised_rrad016

Revised_SupplementaryData_rrad016

## Notes

Presentation at a Conference: The 47th Annual Meeting of the European Radiation Research Society; The 65th Annual Meeting of the Japanese Radiation Research Society; The 64th Annual Meeting of the Japanese Radiation Research Society.
