# Supplementary figures and images for: DAP3-mediated cell cycle regulation and its association with radioresistance in human lung adenocarcinoma cell lines
Source: J Radiat Res. 2023 Apr 6;64(3):520–9. doi: 10.1093/jrr/rrad016 (PMC10214994; doi:10.1093/jrr/rrad016)

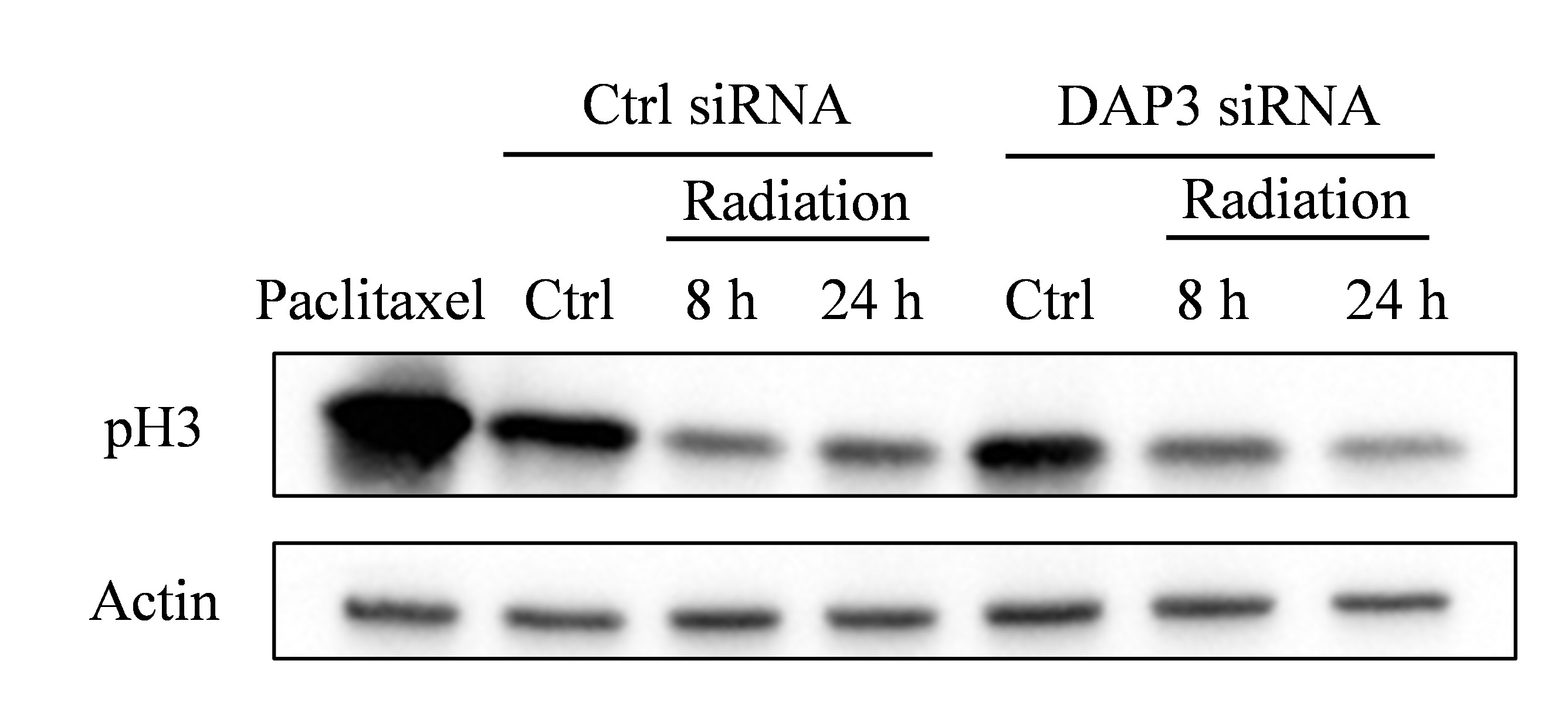

Supplement: Figure_s1_rrad016 [file figure_s1_rrad016.jpeg]

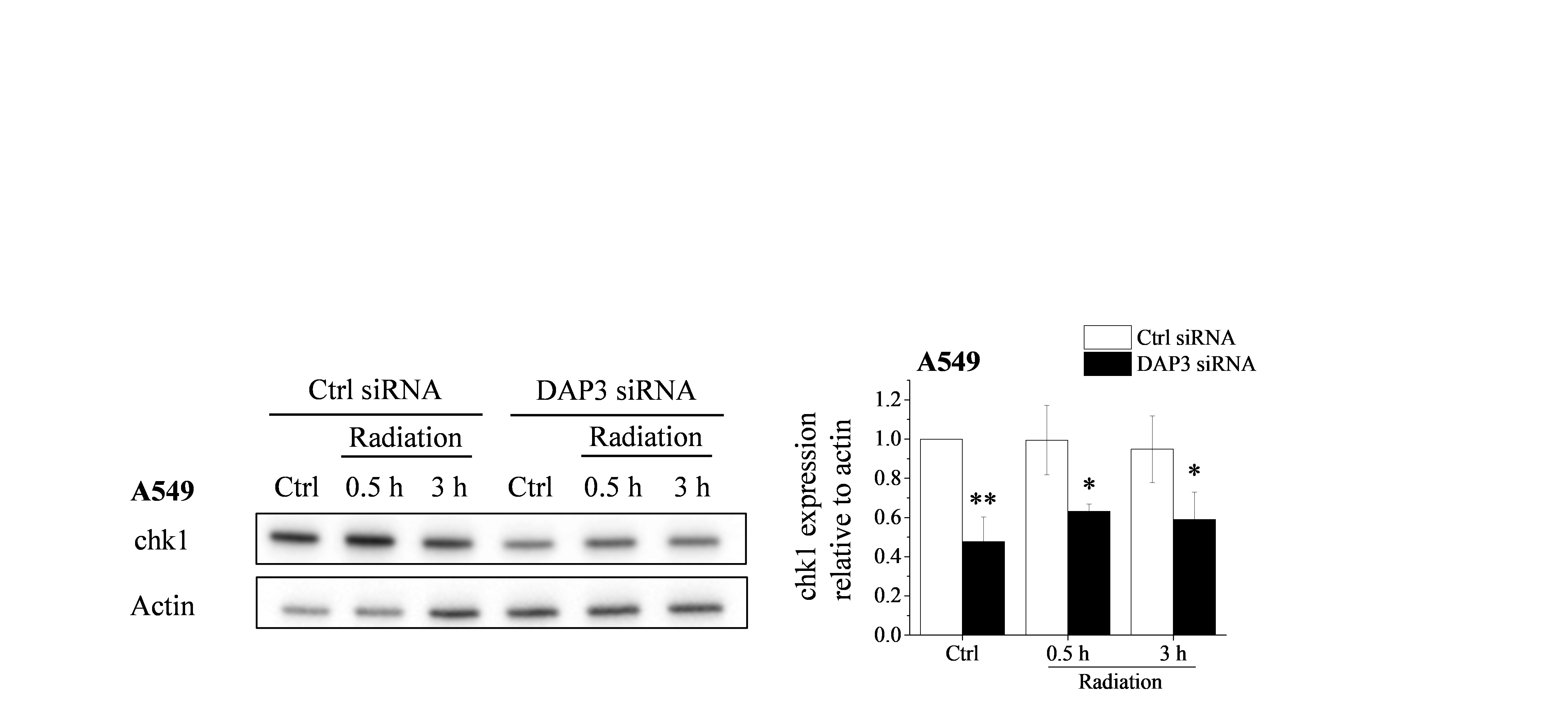

Supplement: Figure_S2_revised_rrad016 [file figure_s2_revised_rrad016.jpeg]

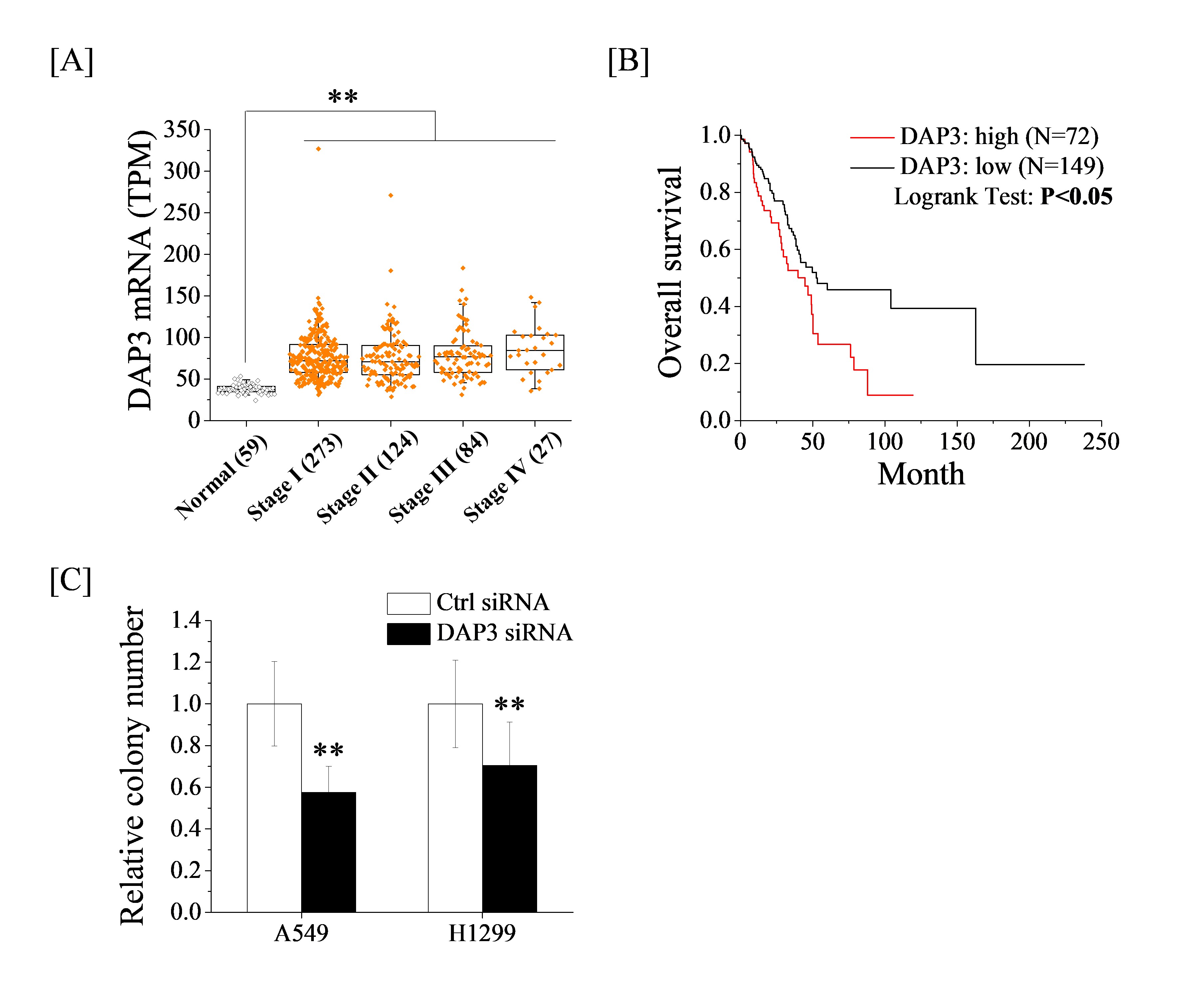

Supplement: Figure_S3_revised_rrad016 [file figure_s3_revised_rrad016.jpeg]
