# Supplementary material for: DAP3-mediated cell cycle regulation and its association with radioresistance in human lung adenocarcinoma cell lines
Source: J Radiat Res. 2023 Apr 6;64(3):520–9. doi: 10.1093/jrr/rrad016 (PMC10214994; doi:10.1093/jrr/rrad016)
Supplement: Revised_SupplementaryData_rrad016 [file revised_supplementarydata_rrad016.docx]

**LEGENDS TO THE SUPPLEMENTARY FIGURES**

**Supplementary Figure 1. Effect of radiation on the expression of the M-phase marker phosphorylated-histone H3.** A549 cells cultured for 24 h in the presence of DMSO or paclitaxel (10 µM), or A549 cells transfected with control or DAP3 siRNA, were treated with radiation and were subsequently cultured for 8 or 24 h. The cells were harvested for the undertaking of Western blot analysis. A representative image of an immunoblot is shown. Actin was used as the loading control, while pH3 indicates the phosphorylated-histone H3.

**Supplementary** **Figure R2. Effects of DAP3 knockdown on the expression of checkpoint kinases 1 in irradiated A549 cells.** A549 cells transfected with control or DAP3 siRNA were treated with 4-Gy irradiation and were cultured for 0.5 or 3 h. The cells were harvested for the undertaking of Western blot analysis. A representative image of an immunoblot is shown. Actin was used as the loading control. The relative values of the chk1/actin ratio are presented. Data are presented as the mean ± SD of three independent experiments. Symbols used: **, *p* < 0.01 *versus* control siRNA.

**Supplementary Figure 3. Association between DAP3 and human LUAD malignancies.** [A] Expression levels of DAP3 mRNA in human LUAD tissues and adjacent normal tissues are shown. Expression data were downloaded from the Cancer RNA-Seq Nexus (http://syslab4.nchu.edu.tw/) and were reanalyzed. [B] The relationship between the overall survival and the DAP3 expression of human LUAD patients in The Cancer Genome Atlas (TCGA) cohorts is shown. cBioPortal for Cancer Genomics (https://www.cbioportal.org/) was used in order to analyze the association between the mRNA levels of DAP3 and the overall survival of human LUAD patients in the TCGA cohort. The patients whose DAP3 mRNA expression z-scores (RNA-Seq V2 RSEM) were greater than 0.5 SD above mean, were defined as “DAP3 high” patients. [C] The clonogenic potential of human LUAD cells transfected with control or DAP3 siRNA was assessed by a colony formation assay. The number of colonies formed by non-irradiated cells transfected with control siRNA was considered as 1.0. Data are presented as the mean ± SD of at least three independent experiments. Symbols used: *, *p* < 0.05, and **, *p* < 0.01; both *versus* control siRNA.
